# Key Brain Network Nodes Show Differential Cognitive Relevance and Developmental Trajectories during Childhood and Adolescence

**DOI:** 10.1523/ENEURO.0092-18.2018

**Published:** 2018-07-11

**Authors:** Knut K. Kolskår, Dag Alnæs, Tobias Kaufmann, Geneviève Richard, Anne-Marthe Sanders, Kristine M. Ulrichsen, Torgeir Moberget, Ole A. Andreassen, Jan E. Nordvik, Lars T. Westlye

**Affiliations:** 1 NORMENT, KG Jebsen Centre for Psychosis Research, Division of Mental Health and Addiction, Oslo University Hospital and Institute of Clinical Medicine, University of Oslo, 0450 Oslo Norway; 2 Sunnaas Rehabilitation Hospital 1453 Bjørnemyr, Norway; 3 Department of Psychology, University of Oslo 0373 Oslo Norway

**Keywords:** adolescence, development, eigenvector centrality, fMRI, graph theory, independent component analysis

## Abstract

Human adolescence is a period of rapid changes in cognition and goal-directed behavior, and it constitutes a major transitional phase towards adulthood. One of the mechanisms suggested to underlie the protracted maturation of functional brain networks, is the increased network integration and segregation enhancing neural efficiency. Importantly, the increasing coordinated network interplay throughout development is mediated through functional hubs, which are highly connected brain areas suggested to be pivotal nodes for the regulation of neural activity. To elucidate brain hub development during childhood and adolescence, we estimated voxel-wise eigenvector centrality (EC) using functional magnetic resonance imaging (fMRI) data from two different psychological contexts (resting state and a working memory task), in a large cross-sectional sample (*n* = 754) spanning the age from 8 to 22 years, and decomposed the maps using independent component analysis (ICA). Our results reveal significant age-related centrality differences in cingulo-opercular, visual, and sensorimotor network nodes during both rest and task performance, suggesting that common neurodevelopmental processes manifest across different mental states. Supporting the functional significance of these developmental patterns, the centrality of the cingulo-opercular node was positively associated with task performance. These findings provide evidence for protracted maturation of hub properties in specific nodes of the brain connectome during the course of childhood and adolescence and suggest that cingulo-opercular centrality is a key factor supporting neurocognitive development.

## Significance Statement

Adolescence is a sensitive period during ontogeny and a maturational gateway to adulthood. A better characterization of the brain changes underpinning cognitive and emotional development occurring during this period is key to inform models of normal and abnormal adaptation. Advanced brain imaging allows for *in vivo* studies of the protracted maturation of brain network function and connectivity during childhood and adolescence. Here, we used functional brain imaging to identify neural hubs during two psychological contexts in 754 children and adolescents. Key regions of the brain network showed increasing importance through adolescence, and individual differences in working memory performance was associated with the centrality of cingulo-opercular network nodes, suggesting a hot spot for neurocognitive development.

## Introduction

The gradual transition from childhood to adulthood is characterized by profound physical, cognitive, and emotional changes. Individual adaptation to the dynamic and socially expanding environment throughout this period is enabled by the large potential for neuroplasticity, e.g., reflected in a transformation of cortical gray matter by a massive synaptic downscaling (Spear, 2000) and increases in brain white matter coherence and volume (Paus, 2005; Westlye et al., 2010; Alnæs et al., 2018). These protracted neurodevelopmental changes form the structural backbone allowing efficient neural signaling, enabling functional integration and adaptation of the brain networks underlying the substantial cognitive and functional maturation seen over this age span (Casey et al., 2008). Of note, while adolescence marks the successful transition from childhood to adulthood for the majority, this is also the period where most mental disorders emerge, supporting the critical role of adolescence as a sensitive period during ontogeny (Paus et al., 2008).

Functional brain connectivity (FC) provides an intriguing window into the developing brain, and it has been extensively studied using resting-state functional magnetic resonance imaging (rs-fMRI). Resting-state networks (RSNs) show high spatial concordance with networks associated with task activations (Smith et al., 2009), and brain connectivity and activation are highly coordinated aspects of brain functioning (Gratton et al., 2016). In a large-scale study on children and adolescents from the Philadelphia Neurodevelopmental Cohort (PNC), Satterthwaite et al. (2014) analyzed fMRI data recorded during the performance of a cognitive task and found that a reciprocal pattern of executive-network activation and default-mode network (DMN) deactivation was associated with working memory performance. This pattern was more predictive of performance differences than activations and deactivations in isolation. Network dynamics and connectivity thus contains complementary information about the neural substrate supporting executive ability and its maturation (Power et al., 2010; Gu et al., 2015).

Whereas the hubs in the default mode and executive-networks are fairly established from five years of age (Hwang et al., 2013; Wu et al., 2013; Cao et al., 2014), key dynamic and functional characteristics of brain nodes display maturational alterations throughout adolescence (Grayson et al., 2014). How these changes support the marked increase in executive abilities during this period is still unclear.

One commonly used metric for investigating hub properties is centrality, which reflects the number and strength of connections of a given node to the rest of the network.

Previous studies have reported a maturational increase in centrality of task positive networks like frontoparietal, cingulo-opercular and task negative DMN nodes (Fransson et al., 2011; Hwang et al., 2013; Cao et al., 2016). While most developmental studies on brain network connectivity have employed rs-fMRI, cognitive engagement modulates the connectivity and centrality of brain nodes (Chadick and Gazzaley, 2011; Cole et al., 2013; Alnæs et al., 2015a,b; Mišić and Sporns, 2016; Kaufmann et al., 2017b). Further, higher cognitive load has been shown to increase the sensitivity for age-related differences in FC (Dørum et al., 2017), motivating investigation of brain dynamics beyond the unconstrained resting state.

Here, to characterize age-related differences in functional connectivity in the adolescent brain, we estimated whole-brain voxel-wise eigenvector centrality (EC) of fMRI data collected during resting state and a working memory task (fractal *n*-back) for 754 children and adolescents aged 8–22 years from the PNC (Satterthwaite et al., 2014). To allow for multiple levels of inference and to investigate the hierarchical organization of the brain network nodes, we decomposed the voxel-wise EC maps using independent component analysis (ICA) and tested for main effects of task, age, and their interactions both on the voxel-wise EC maps and the ICA subject weights. Based on previous studies and models of adolescent brain development, we hypothesized (1) increased centrality in task positive networks and decreased centrality in DMN, sensorimotor, and visual networks in response to task engagement; (2) and that these centrality changes would be associated with task performance. Furthermore, (3) we hypothesized that these effects on network centrality would parallel task-related activation through a characteristic pattern of increased activation in networks involved in task engagement and decreased activation in DMN. Lastly, we hypothesized (4) that the centrality of key nodes would show evidence of protracted maturation during the sampled age-span, with differential age-related differences across nodes and conditions, possibly indicating increasing age-dependent differentiation between the two psychological contexts for both activation and centrality.

## Materials and Methods

### Sample and exclusion criteria

The analysis was performed on the publicly available PNC (Satterthwaite et al., 2014), access permission number 8642. All participants gave written informed consent, and the study was approved by the review boards of the University of Pennsylvania and the Children’s hospital of Philadelphia. After exclusion of participants with severe medical and psychiatric conditions, missing task responses, and poor normalization of fMRI data, the final sample comprised 754 individuals aged 8–22 years (405 males, mean 15 years old, SD 3.3 years). All subjects were recruited through the Center for Applied Genomics at The Children’s Hospital in Philadelphia.

### MRI acquisition

MRI scans were acquired at the University of Pennsylvania on a 3T Siemens TIM Trio scanner. An anatomic scan used here for registration purposes was acquired using a 3D T1-weighted magnetization prepared rapid acquisition gradient echo (MPRAGE) sequence (TR: 1.81 s, TE: 3.5 ms, FA: 9°, FOV: 240 × 180 mm, slice thickness: 1 mm, slices: 160). Functional images were acquired using single-shot, interleaved multislice, gradient-echo, echo planar imaging (GE-EPI) sequence (TR: 3.0 s, TE: 32 ms, FA: 90°, FOV: 192 mm^2^).

### fMRI task paradigm and behavior

The task has been previously described in detail (Satterthwaite et al., 2013). Briefly, we included data from two fMRI runs: One was a resting-state run (eyes open while fixating on central fixation cross), while the other was collected while participants performed a fractal version of the *n*-back task. The *n*-back task consisted of three load levels: 0-back, 1-back, and 2-back. During 0-back, participants were asked to press a button every time a target fractal was presented. During 1- and 2-back, participants were asked to respond whenever the presented fractal was identical to the one presented one or two trials prior, respectively. Each run consisted of three blocks per condition, and each block consisted of 20 trials, of which five were targets and 15 were non-target fractals. We computed the total hit-rate, false-positive rate, and D-prime. The latter was calculated by subtracting *z* score for false positives from the *z* score for hit-rate from the in-scanner responses during the *n*-back task (Stanislaw and Todorov, 1999).

### fMRI data processing

fMRI data were processed using FMRI Expert Analysis Tool (FEAT) version 6.00, from FMRIB’s Software Library (FSL; Smith et al., 2004; Jenkinson et al., 2012), and included the following steps: correction for motion using MCFLIRT (Jenkinson et al., 2002), linear trend removal and high-pass filtering (0.01 Hz), removal of non-brain tissue using BET (Smith, 2002), spatial smoothing with a Gaussian kernel of full width at half maximum (FWHM) of 6 mm (SUSAN; Smith and Brady, 1997). We employed automated procedures for data denoising, including (FIX; Salimi-Khorshidi et al., 2014) and ICA-AROMA (Pruim et al., 2015), which has been reported to reduce risk of inflating age-related effects due to higher in-scanner head-movement (Kaufmann et al., 2017a). Further, we applied nonlinear registration using FNIRT to Montreal Neurologic Institute (MNI) 152 standard space using the T1-weighted scan as an intermediate.

### EC mapping (ECM)

Following previous studies (Alnæs et al., 2015b; Skåtun et al., 2016), we first created a group mask containing common voxels across all participants and sessions (*n*-back and rest). To accommodate images to EC estimation, the pre-processed and normalized *n*-back, resting state runs and analysis mask were resampled to an isotropic voxel resolution of 3 mm, converted to the Vista image format (Pope and Lowe, 1994) using LIPSIA (Lohmann et al., 2001) and submitted to estimation of voxel-wise EC (Lohmann et al., 2010) using the absolute of the full correlation coefficients (equal weight to positive and negative correlations), yielding one EC map per run for each participant. Individual EC maps and brain masks were converted to nifti format, resampled to 2-mm isotropic resolution, and submitted to further analysis (ICA and voxel-wise statistics).

### ICA on EC maps

The 1508 EC maps were decomposed into a fixed set of 40 components, for optimal trade-off between spatial resolution of components and not over-fitting the model, using spatial ICA in MELODIC (Beckmann and Smith, 2004). To assess effects of condition on EC for each IC, the associated subject-weights from the ICA was used to calculate the difference in subject weights between rest and *n*-back runs for each IC. For visualization of the network structure, we used subject weights for both *n*-back and rest to form a component by component correlation matrix, which was submitted to hierarchical clustering using FSLNets (Smith et al., 2011). ICs covering white matter and CSF were excluded before clustering.

### Dual regression

To assess the relationships between the centrality and task-related activation of the ECM-based ICs, we extracted individual time series from each of the component spatial maps from the *n*-back data using dual regression (Nickerson et al., 2017). GLMs were estimated for each participant and each component’s time series, modeling 0-back, 1-back, and 2-back as well as responses and instructions. As a measure of BOLD task effect, β-estimates for 0-back was subtracted from the average of 1-back and 2-back β-estimates.

### Voxel-wise analysis

For transparency, we compared our main results obtained using ICA-based decomposition of EC maps with voxel-wise effects of task and age and their interactions on EC using FSL randomize (Winkler et al., 2014). First, we computed difference maps by subtracting the individual resting-state EC maps from the *n*-back EC maps. We tested for main effect of condition by performing a one-sample *t* test on the difference maps (*n*-back minus rest). We further tested for associations with age by performing linear regression on rest, *n*-back, and the difference maps, including sex as covariate. Statistical inference was done by permutation testing with 5000 iterations and threshold-free cluster enhancement (Smith and Nichols, 2009).

### Statistical analysis

Analyses beyond the voxel- and imaging domain were performed in MATLAB 2014a (MathWorks). To test for effects of age on EC, we performed linear regressions with the subject weights from *n*-back or resting-state runs as dependent variable, and age and sex as independent variables for all ICs. To test for main effect of task on each component, we performed one-sample *t* tests on the difference scores between rest and *n*-back weights. To test for interactions between task and age on EC, we performed linear regressions with the difference score as dependent variable and age and sex as independent variables. To test for associations between EC and task performance (hit-rate, false positives, and D-prime), we used the same derived difference score as dependent variable, and hit-rate, false positives, or D-prime as independent variables in separate analyses, in addition to age and sex.

In the same manner, using the β-estimates derived from dual regression on the *n*-back data, we tested for effect of task by performing one-sample *t* tests. To test for effect of age, we performed linear regression with task effect as the dependent variable and age and sex as independent variables.

To test for associations between task-related effects on centrality and activation for each IC, we correlated the difference EC-score (*n*-back minus rest) with β-estimates for task engagement. To account for multiple comparisons, we adjusted the false discovery rate (FDR, q = 0.05) across all tests and components (Genovese et al., 2002). For visualization of the association between centrality and activation, component-wise EC-difference-weights and β-estimates for the *n*-back task was imported to R (http://www.r-project.org) and plotted with the ggplot2-package (Wickham, 2009).

## Results

### ICA and hierarchical clustering

**Figure 1.**
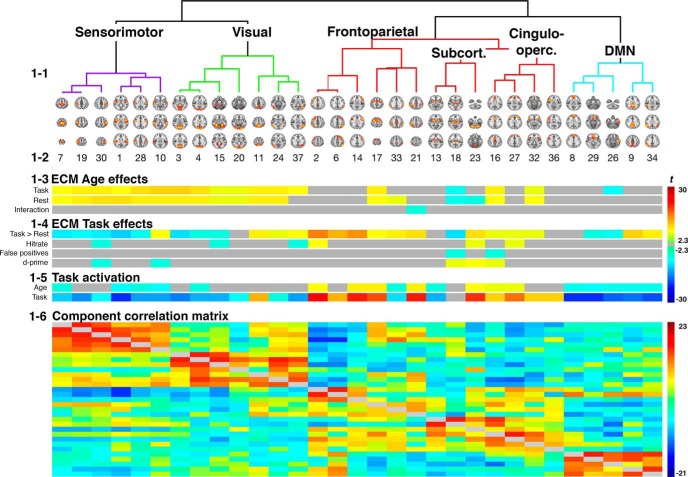
Results from the ICA analysis and corresponding statistics. (1-1) The hierarchical clustering of the components, based on the partial correlations between the difference weights (*n*-back minus rest). (1-2) Visualization of the IC spatial maps. (1-3) Visualization of statistics reflecting the effect of age on EC during both *n*-back and rest, and the interaction between age and task engagement respectively. (1-4) The effect of task engagement on EC for the ICs, and effect of EC on hit-rate, false positives and d-prime. (1-5) The significant effects of age on task activation, and the effect of task engagement. Summary of statistics for task effect is displayed in table 2, age effects in table 3. (1-6) The IC-subject weight correlation-matrix. Correlations across both conditions are displayed below the diagonal and was used for the hierarchical clustering displayed in 1-1. Above the diagonal are the correlations between IC difference-weights (*n*-back minus rest).

ICA of EC maps yielded 31 spatial maps corresponding to cortical gray-matter components, including frontoparietal, default mode, visual networks, and nine maps corresponding to white matter and CSF (for an anatomic description of the 31 maps included in the hierarchical clustering, see Table 1). Figure 1,1-1 shows the hierarchical clustering based on the correlation between IC-weights across *n*-back and rest, and Figure 1,1-2 shows the associated spatial maps. Clustering revealed a structure coherent with known large-scale functional networks, largely reflecting visual, sensorimotor, frontoparietal, subcortical, cingulo-opercular, and DMN.

**Table 1.** Anatomic location for each IC

Sensorimotor components	
IC	Area
7	Bilateral superior pre/postcentral gyrus
19	Right pre/postcentral gyrus, left superior cerebellum
30	Left pre/postcentral gyrus, right superior cerebellum
1	Bilateral juxtapositional cortex, bilateral insular cortex, bilateral central opercular cortex
28	Bilateral precentral gyrus
10	Bilateral superior temporal gyrus
Visual components	
IC	Area
3	Bilateral intracalcarine cortex, bilateral cuneal cortex, bilateral lingual gyrus
4	Bilateral lateral superior occipital cortex
15	Bilateral occipital fusiform gyrus, bilateral lingual gyrus
20	Bilateral occipital pole
11	Bilateral precuneus cortex
24	Right lateral occipital cortex
37	Left lateral occipital cortex
Frontoparietal-associated components	
IC	Area
2	Right superior parietal/lateral occipital cortex, right middle frontal gyrus, right paracingulate gyrus
6	Left superior parietal/lateral occipital cortex, left middle frontal gyrus, left paracingulate gyrus
14	Left middle frontal/inferior frontal gyrus
17	Bilateral superior frontal gyrus, bilateral juxtapositional cortex
33	Bilateral superior posterior cingulate/precuneus, bilateral supramarginal gyrus
21	Bilateral superior parietal lobe/precentral gyrus/posterior cingulate/medial prefrontal cortex
Subcortical components	
13	Bilateral putamen
18	Bilateral thalamus
23	Cerebellum
Cingulo-opercular associated components	
16	Anterior cingulate cortex
27	Bilateral frontal pole
32	Bilateral insula, bilateral frontal operculum
36	Right Insular cortex/frontal operculum
DMN-associated components	
IC	Area
8	Bilateral superior medial prefrontal cortex
29	Bilateral inferior medial prefrontal cortex
26	Left middle temporal gyrus
9	Bilateral posterior cingulate cortex
34	Right posterior cingulate cortex, bilateral lateral occipital cortex

### Component-wise EC task effects

Task engagement (*n*-back > rest) was associated with distributed differences in EC across most nodes. Figure 1,1-4 displays significant (FDR corrected, *q* = 0.05) associations between EC, task engagement and task performance, and Table 2 summarizes corresponding statistics. All frontoparietal components displayed a significant increase in EC with task engagement, and for one of the components (IC 2) higher centrality was significantly associated with better performance. Three of the four cingulo-opercular nodes displayed significant increase of centrality with task engagement, and higher EC was associated with better performance (two of four ICs). The subcortical components displayed a mixed pattern of centrality changes, thalamus decreased, but cerebellum increased with task engagement. Higher EC for both cerebellum and thalamus was associated with better performance. DMN components displayed both increase (two of five ICs) and decrease (two of five ICs) in centrality with task engagement, but no significant associations with task performance. Sensorimotor components displayed reduced centrality (five of six ICs) with task engagement, and for two of the components, the degree of reduction was associated with task performance. Visual components displayed a mixed pattern with both increased (three of seven ICs) and decreased (three of seven ICs) centrality with task engagement, and centrality in two components showed negative correlations with task performance.

**Table 2.** Effect of task engagement on EC (*n*-back minus rest) and effect of EC on hit-rate, false positives, and D-prime, and effect of task on activation

IC	Task effect		Hit-rate		False positives		d-prime		Activation	
	*t*	*p*	*t*	*p*	*t*	*p*	*t*	*p*	*t*	*p*
Sensorimotor										
7	**-7.95**	**6.7E-15**	-0.2	8.4E-01	0.99	3.2E-01	-0.26	8.0E-01	**-11.82**	**1.1E-29**
19	**-6.46**	**1.9E-10**	-1.58	1.2E-01	-0.21	8.4E-01	-1.09	2.8E-01	**-17.46**	**1.3E-57**
30	**-6.65**	**5.6E-11**	**-3.00**	**2.8E-03**	1.53	1.3E-01	**-3.48**	**5.4E-04**	**-7.10**	**2.8E-12**
1	**-9.73**	**3.6E-21**	-1.37	1.7E-01	0.88	3.8E-01	-1.92	5.5E-02	**-26.91**	**2.7E-112**
28	**-3.96**	**8.0E-05**	-0.22	8.3E-01	1.25	2.1E-01	-0.13	8.9E-01	**-15.77**	**1.3E-48**
10	**4.26**	**2.3E-05**	-1.95	5.2E-02	0.38	7.0E-01	**-2.32**	**2.1E-02**	**-16.49**	**2.2E-52**
Visual										
3	**-9.49**	**2.9E-20**	2.08	3.8E-02	-0.76	4.5E-01	1.88	6.1E-02	**-18.66**	**3.7E-64**
4	**-3.53**	**4.4E-04**	0.36	7.2E-01	-0.85	4.0E-01	1.29	2.0E-01	**-15.29**	**3.6E-46**
15	**-2.9**	**3.9E-03**	**-2.33**	**2.0E-02**	1.44	1.5E-01	-2.02	4.4E-02	**-15.35**	**1.8E-46**
20	-0.17	8.6E-01	1.09	2.7E-01	-0.46	6.4E-01	1.19	2.3E-01	**-6.81**	**2.1E-11**
11	**6.45**	**2.0E-10**	-0.12	9.0E-01	-1.24	2.1E-01	1.12	2.6E-01	**13.28**	**2.6E-36**
24	**2.36**	**1.9E-02**	-1.05	3.0E-01	0.07	9.4E-01	-0.45	6.5E-01	**-3.47**	**5.5E-04**
37	**4.37**	**1.4E-05**	**-2.45**	**1.5E-02**	1.12	2.6E-01	-1.91	5.6E-02	**-15.82**	**7.1E-49**
Frontoparietal										
2	**16.09**	**2.8E-50**	**3.63**	**3.1E-04**	-0.8	4.2E-01	2.21	2.8E-02	**29.80**	**1.7E-129**
6	**12.14**	**4.2E-31**	1.04	3.0E-01	-0.2	8.4E-01	0.33	7.4E-01	**13.19**	**6.8E-36**
14	**15.21**	**9.3E-46**	1.26	2.1E-01	-0.84	4.0E-01	0.79	4.3E-01	**26.79**	**1.4E-111**
17	**4.68**	**3.4E-06**	1.6	1.1E-01	-1.6	1.1E-01	1.36	1.7E-01	**21.79**	**5.1E-82**
33	**6.35**	**3.8E-10**	0.22	8.3E-01	-1.3	1.9E-01	0.35	7.3E-01	**-9.75**	**3.1E-21**
21	**6.58**	**8.5E-11**	-1.36	1.7E-01	-0.69	4.9E-01	-1.47	1.4E-01	**28.01**	**7.8E-119**
Subcortical										
13	-1.21	2.3E-01	-0.22	8.2E-01	-1.37	1.7E-01	0.81	4.2E-01	**-14.82**	**8.7E-44**
18	**-8.51**	**9.4E-17**	0.75	4.5E-01	**-2.76**	**6.0E-03**	**2.4**	**1.7E-02**	-1.81	7.0E-02
23	**6.74**	**3.2E-11**	**2.69**	**7.3E-03**	-1.96	5.0E-02	**3.7**	**2.3E-04**	**25.37**	**3.9E-103**
Cingulo-opercular										
16	**2.78**	**5.6E-03**	2.19	2.9E-02	**-2.36**	**1.8E-02**	**2.32**	**2.1E-02**	**9.57**	**1.6E-20**
27	**3.33**	**9.1E-04**	**2.64**	**8.5E-03**	0.4	6.9E-01	1.91	5.6E-02	**19.83**	**9.6E-71**
32	**3.11**	**1.9E-03**	1.29	2.0E-01	-1.53	1.3E-01	0.97	3.3E-01	**9.35**	**9.9E-20**
36	1.99	4.7E-02	0.73	4.6E-01	-0.96	3.4E-01	0.58	5.7E-01	**9.15**	**5.4E-19**
DMN										
8	1.73	8.4E-02	-0.7	4.8E-01	-0.28	7.8E-01	-0.46	6.5E-01	**-27.31**	**1.2E-114**
29	**-3.37**	**7.8E-04**	-0.18	8.6E-01	1.42	1.6E-01	-0.89	3.8E-01	**-33.81**	**3.6E-153**
26	**-4.03**	**6.1E-05**	-1.38	1.7E-01	1.77	7.7E-02	-1.53	1.3E-01	**-22.76**	**1.2E-87**
9	**9.03**	**1.4E-18**	0.74	4.6E-01	-1.71	8.7E-02	1.11	2.7E-01	**-21.41**	**8.6E-80**
34	**6.51**	**1.3E-10**	-1.09	2.8E-01	-0.1	9.2E-01	-1.26	2.1E-01	**-23.77**	**1.3E-93**

Significant results are highlighted. All results are corrected for multiple comparisons (FDR), *q* = 0.05.

### Component-wise activation

Significant effects (FDR corrected, *q* = 0.05) of task engagement are displayed in Figure 1,1-5, lower panel. Table 2 summarizes the corresponding statistics. The component-wise activation pattern revealed several similarities with the pattern for EC. Task engagement was associated with significantly increased activation in the frontoparietal (six of seven ICs) and cingulo-opercular (four of four ICs) components. Among the subcortical components, we found increased activation in cerebellum and decreased activation in putamen. The visual, sensorimotor, and DMN components displayed reduced activation during task.

### Associations between centrality and activation

**Figure 2.**
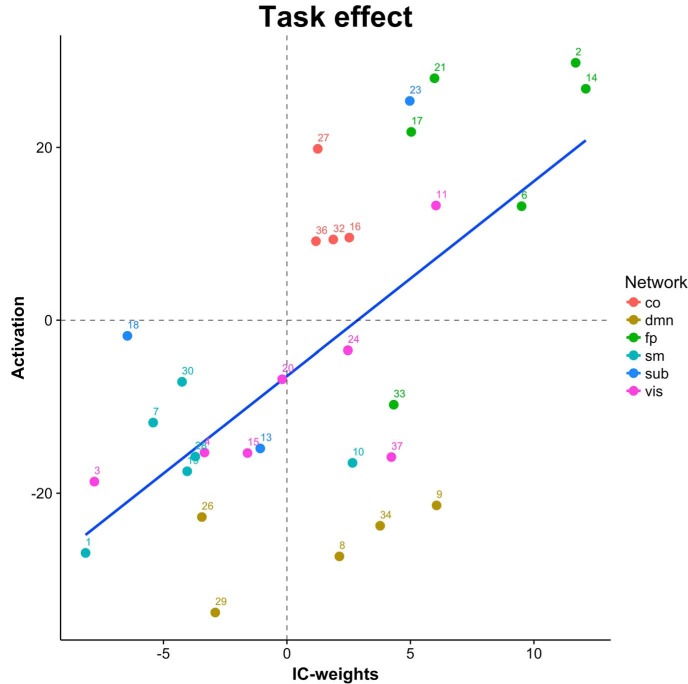
Associations between BOLD and EC changes due to task engagement. co: cingulo-opercular network; dmn: default**-**mode network; fp: frontoparietal; sm: sensorimotor; sub: subcortical; vis: visual.

Overall, we found a positive correlation between activation and EC (*r* = 0.64, *p* = 1.11e^−4^): components showing increased activity with task generally also showed task-related increases in EC. Figure 2 displays the component-wise association between task-related activation and differences in EC between *n*-back and rest. However, the results revealed some notable exceptions. For example, whereas components in the frontoparietal cluster showed both strong task-related activation and increased centrality, and visual and sensorimotor cluster components showed deactivation and decreased centrality, DMN components showed overall task related deactivations, but a mixed pattern of increased and decreased centrality.

### Component-wise associations with age

**Figure 3.**
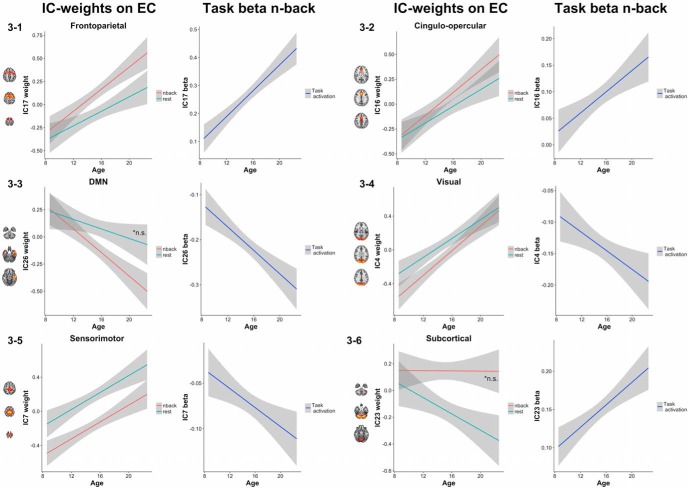
Age effect on EC during both *n*-back and rest on the left panel, and activation in different network nodes on the right panel. (3-1) Frontoparietal, (3-2) cingulo-opercular, (3-3) DMN, (3-4) visual, (3-5) sensorimotor, and (3-6) subcortical. Nonsignificant associations are annotated.

Figure 1, 1-3 and 1-5, upper panel, shows the significant (FDR corrected, *q* = 0.05) associations between age centrality and task-related activation, whereas Figure 3 shows the age-associated trajectory for selected components. Table 3 summarizes the corresponding statistics. For frontoparietal components, we found significant positive associations between centrality and age in one component during *n*-back and two components during rest. One of the frontoparietal components displayed a significant interaction effect suggesting a stronger negative age effect during rest compared to *n*-back. In contrast, we found significantly increased activation with increasing age across all frontoparietal components, with the exception of one. Cingulo-opercular components displayed significant age-related increases in EC in two of four components, anterior cingulate and insula respectively. The increase in EC was paralleled by significant increase in activation in all cingulo-opercular components. Among the subcortical components, thalamus and cerebellum showed significant age-related decreased centrality, and the latter also showed age-related increased activation. Within the DMN cluster, one component showed significant age-related decrease in EC during *n*-back. In contrast, all DMN-components showed significant decrease in activation with increasing age, indicating stronger deactivation with higher age. Visual and sensorimotor components displayed a highly similar pattern: age-related increases in EC during both task and rest, but decreased activation with higher age. The exception was one component, showing age-related increases both in centrality and activation.

**Table 3.** Effect of age on EC in *n*-back, rest, and the interaction between *n*-back and age, and effect of age on activation

IC	Age *n*-back		Age rest		Task × age		Activation age	
	*t*	*p*	*t*	*p*	*t*	*p*	*t*	*p*
Sensorimotor								
7	**4.71**	**2.90E-06**	**4.48**	**8.50E-06**	-0.05	9.60E-01	**-2.89**	**4.0E-03**
19	**6.12**	**1.50E-09**	**5.46**	**6.40E-08**	0.77	4.40E-01	-1.02	3.1E-01
30	**6.15**	**1.30E-09**	**7.00**	**5.70E-12**	-0.59	5.60E-01	0.51	6.1E-01
1	**4.98**	**8.10E-07**	**7.58**	**1.00E-13**	-0.96	3.40E-01	**-5.34**	**1.2E-07**
28	**7.14**	**2.20E-12**	**6.14**	**1.40E-09**	0.42	6.70E-01	**-3.54**	**4.2E-04**
10	**8.49**	**1.10E-16**	**5.8**	**9.90E-09**	2.17	3.00E-02	**-5.22**	**2.3E-07**
Visual								
3	**8.49**	**1.10E-16**	**6.39**	**2.80E-10**	1.68	9.20E-02	-0.19	8.5E-01
4	**6.74**	**3.10E-11**	**5.15**	**3.30E-07**	1.28	2.00E-01	**-2.56**	**1.1E-02**
15	**3.4**	**7.20E-04**	**3.52**	**4.60E-04**	-0.05	9.60E-01	-0.39	7.0E-01
20	**2.93**	**3.50E-03**	**3.31**	**9.80E-04**	0.11	9.10E-01	1.58	1.1E-01
11	**6.48**	**1.70E-10**	**6.39**	**2.90E-10**	0.07	9.40E-01	1.73	8.4E-02
24	**6.3**	**4.90E-10**	**5.29**	**1.60E-07**	0.02	9.80E-01	0.08	9.4E-01
37	**4.3**	**1.90E-05**	1.93	5.40E-02	1.79	7.40E-02	-1.05	2.9E-01
Frontoparietal								
2	1.25	2.10E-01	0.42	6.70E-01	0.88	3.80E-01	**4.62**	**4.6E-06**
6	-1.45	1.50E-01	0.67	5.00E-01	-1.67	9.60E-02	1.09	2.8E-01
14	-0.09	9.30E-01	-0.9	3.70E-01	0.6	5.50E-01	**5.73**	**1.5E-08**
17	**5.53**	**4.30E-08**	**3.61**	**3.20E-04**	1.26	2.10E-01	**6.40**	**2.7E-10**
33	1.9	5.80E-02	**2.64**	**8.40E-03**	-0.69	4.90E-01	**-2.55**	**1.1E-02**
21	-1.61	1.10E-01	1.46	1.50E-01	**-2.36**	**1.90E-02**	**5.44**	**7.2E-08**
Subcortical								
13	-0.77	4.40E-01	-1.88	6.10E-02	0.95	3.40E-01	-2.33	2.0E-02
18	**-3.65**	**2.80E-04**	**-4.57**	**5.70E-06**	0.99	3.20E-01	-0.37	7.1E-01
23	0.06	9.60E-01	**-2.49**	**1.30E-02**	2.26	2.40E-02	**4.15**	**3.6E-05**
Cingulo-opercular								
16	**5.14**	**3.50E-07**	**3.81**	**1.50E-04**	1.08	2.80E-01	**3.38**	**7.6E-04**
27	-0.38	7.10E-01	1.44	1.50E-01	-1.38	1.70E-01	**3.34**	**8.8E-04**
32	**2.4**	**1.70E-02**	**3.8**	**1.60E-04**	-0.92	3.60E-01	1.27	2.0E-01
36	1.04	3.00E-01	1.43	1.50E-01	-0.32	7.50E-01	1.82	6.9E-02
DMN								
8	2.24	2.50E-02	0.6	5.50E-01	1.26	2.10E-01	**-4.73**	**2.7E-06**
29	-1.83	6.70E-02	-0.22	8.20E-01	-1.23	2.20E-01	**-5.62**	**2.7E-08**
26	**-5.36**	**1.10E-07**	**-2.1**	**3.60E-02**	-2.23	2.60E-02	**-4.65**	**4.0E-06**
9	0.03	9.70E-01	-0.87	3.80E-01	0.65	5.10E-01	**-4.50**	**7.8E-06**
34	1.1	2.70E-01	-0.01	9.90E-01	0.82	4.10E-01	**-4.34**	**1.6E-05**

Significant results are highlighted. All results are corrected for multiple comparisons (FDR), *q* = 0.05.

### Voxel-wise analysis

**Figure 4.**
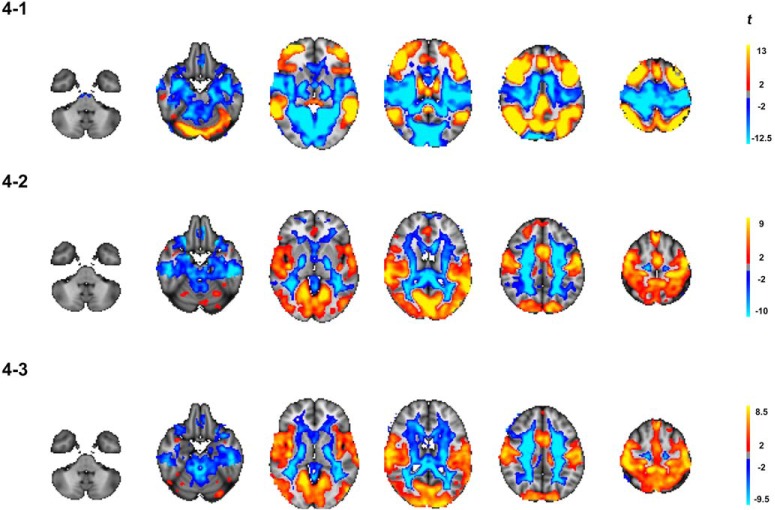
Results from full-brain voxel-wise analysis. (4-1) Significant changes in EC associated with differentiation between task and rest across the age-span. (4-2) Age-effect in the *n*-back data and (4-3) Age-effect in the rest-data. All maps were corrected for multiple comparisons using permutation testing (*n* = 5000) and TFCE with an alpha of 0.05 (FWE, two-tailed). Only significant results are displayed.

Figure 4 shows the results from the full-brain voxel-wise analyses. Figure 4,4-1 displays the difference in centrality during *n*-back compared to rest, suggesting higher centrality in frontoparietal areas and posterior DMN and lower centrality in sensorimotor areas, mirroring the ICA-based analysis. Figure 4,4-2 and 4-3, shows associations between centrality and age during *n*-back and rest, respectively. We found significant age-related increases in centrality in both conditions in visual and sensorimotor regions as well as in the anterior cingulate. During *n*-back, we found significant age-related differences in the frontal pole, extending to the superior frontal gyrus. Negative associations with age were found in large white matter and CSF regions. However, there was no significant age effect in the derived difference maps, indicating no evidence of interactions between age and condition.

## Discussion

Using graph-based metrics and data-driven decomposition of fMRI data obtained during an unconstrained resting state condition and performance of an *n*-back task, the current study yielded three main findings. First, we demonstrated the sensitivity of EC to differences in task demands. Next, we revealed age-dependent alterations in the centrality of key brain networks, with increasing centrality in sensorimotor, visual, and cingulo-opercular network components and decreasing centrality in thalamus, cerebellum, and left temporal gyrus. Finally, the lack of condition by age interactions suggests that these age-related differences in network centrality are not strongly dependent on mental state, supporting that the maturation of the functional architecture of the brain is relatively pervasive across psychological contexts. These main findings will be discussed below.

### Brain network centrality and task engagement

The most prominent finding in the current study was increased centrality of frontoparietal, cerebellar and cingulo-opercular components, and decreased centrality in sensorimotor and thalamic components with task engagement, which align with previous investigations of EC in adults (Alnæs et al., 2015b; Hearne et al., 2017). FC during task engagement is associated with increased cross-network connectivity, and decreased network modularity (Di et al., 2013; Cole et al., 2014; Shine et al., 2016). This dynamic shift in connectivity pattern in response to task demands supports increased global effectiveness, through shortening path lengths in the network, and increased efficiency of information transfer across the global network (Stanley et al., 2015). These alterations in brain network dynamics enable flexible reconfiguration in response to task demands, which supports executive control, including working memory performance (Braun et al., 2015; Stanley et al., 2015). Among the proposed neurocognitive mechanisms is an increased influence of executive nodes exerting top down control (Chadick and Gazzaley, 2011), as well as suppression of task-irrelevant nodes to minimize interference which may be detrimental to performance (Tomasi et al., 2014). Indeed, within-network visual and sensorimotor connectivity have been reported to decrease with task demand, with simultaneous increases in connectivity with frontoparietal and cerebellar nodes (Kellermann et al., 2012; Spadone et al., 2015; Brissenden et al., 2016; Kwon et al., 2017), which facilitates both suppression and biasing of sensory input. Thalamus plays a key role in the gating of sensory input toward higher cortical network structures (Halassa and Kastner, 2017; Hwang et al., 2017). Interestingly, reduced connectivity between specific subclusters of the thalamus and visual and motor cortices in response to task demands has been reported, also in absence of altered FC with executive or DMN networks (Fan et al., 2015). In light of these observations, our findings indicate that task engagement reduces thalamic FC with cortical areas, facilitating the suppression and filtering of sensory signal during task engagement.

Our results align with previous findings highlighting the importance of increased influence of executive and cerebellar nodes during task engagement, as higher cingulo-opercular, cerebellar and frontoparietal centrality was associated with better task performance, and higher centrality in visual and sensorimotor nodes was associated with lower performance. Thalamus in particular was further associated with decrease in centrality during task engagement reflecting a drop in global FC, and the degree of differentiation between task and rest EC was positively associated with performance. Indeed, our results support both the interference hypothesis (D’Esposito et al., 1999), suggesting that performance during the *n*-back task is supported by increased influence from frontoparietal and cingulo-opercular nodes, providing top-down attentional control, as well as the sensory gating hypothesis (Cromwell et al., 2008), reflected through the decreased thalamic EC, which was associated with better performance.

Notably, to a large extent, the centrality pattern observed during task engagement paralleled the observed activation patterns. Studying activation and connectivity across several cognitive tasks, Gratton et al. (2016) reported coordinated changes in connectivity and activation, in which an increase in both measures were observed for brain regions involved in active task control. This supports the notion of connectivity and activation as coordinated aspects of brain functioning. Overall, our results comply with these findings of coordinated activity and network changes for the frontoparietal and the cingulo-opercular-network. However, while the DMN displayed a task-related increase in centrality, it also displayed the commonly observed deactivation in response to task demands. This could reflect a coordinated disengagement, as previous studies have reported task-driven increases in internal DMN connectivity and coherence during cognitive engagement (Anticevic et al., 2012; Goparaju et al., 2014). On the other hand, recent studies using a graph theoretical approach alternatively suggest that the DMN dynamically changes its cross talk with other networks during task execution (Vatansever et al., 2015a,b) and displays larger integration with the full connectome. In particular, variability in functional connectivity between executive networks and DMN during an executive task was shown to be positively associated with performance (Douw et al., 2016). Taken together, there is still a large degree of uncertainty regarding DMN involvement during task engagement, and further studies are needed to gain a better understanding of the DMN dynamics and its implications for brain development and cognitive functioning.

### Age-related differences in activation

In line with our expectations and previous activation analysis in an overlapping sample (Satterthwaite et al., 2013), our analysis revealed age-related increases in the activation of frontoparietal components and increased deactivation of visual and DMN components, suggesting increased differentiation of these networks with age in response to task demands. We also observed additional age-related effects in cingulo-opercular and subcortical components, which were not reported earlier. This discrepancy may be related to higher sensitivity for the current ICA-based approach compared to the voxel-wise analysis, allowing for higher component-wise specificity and effectively reducing number of comparisons.

### Age-related differences in centrality

For the connectivity and graph-based analysis, we found increasing centrality with increasing age in key cingulo-opercular components, as well as posterior cingulate, and superior frontal gyrus, reflecting increased centrality in task positive visual and sensorimotor components, along with reductions in thalamus, cerebellum and temporal gyrus during both *n*-back and rest. We found no significant interaction effects between task and age. Although one should be cautious when interpreting null findings, the lack of significant interactions suggests that age-related differences in network characteristics are not strongly dependent on cognitive load. A recent large-scale developmental study demonstrated that maturation of the structure and organization of the brain functional connectome is characterized by increasing segregation of large-scale functional networks, along with differentiation of the various networks’ influence on the full connectome (Gu et al., 2015). Indeed, converging evidence points toward an increasing global influence from DMN in general, and cingulo-opercular network nodes during cognitive processing, as hallmarks of healthy development (Gu et al., 2015; Marek et al., 2015). Specifically, Marek et al. (2015) reported that maturational increase in inhibitory control during early adolescence is accompanied by increased between-network connectivity, in particular within the sensorimotor, visual, and cingulo-opercular networks. Furthermore, temporal variability in cingulo-opercular connectivity has been suggested to serve as a proxy for brain maturation (Sato et al., 2015b).

The importance of the cingulo-opercular network for cognitive functioning is further supported by studies investigating both healthy adults and clinical groups. Cingulo-opercular activity in healthy adults is associated with the ability to maintain sustained attention and integrate feedback (Cocchi et al., 2013), and within-network efficiency measured as degree of edge density has been linked to cognitive flexibility (Sheffield et al., 2016). Complementary, decreased functional connectivity between cingulo-opercular, sensorimotor, and DMN nodes with aging has been linked to deteriorated cognitive performance (Meier et al., 2012), and lesions targeting hub cingulo-opercular nodes are associated with greater cognitive impairment compared to non-hub nodes (Warren et al., 2014). Indeed, structural abnormalities in hubs has been found to be overrepresented across a range of brain disorders (Crossley et al., 2014), and abnormalities in anterior cingulate of the cingulo-opercular network was proposed as one of the key brain predictors for schizophrenia. Identifying deviant hub development may show promise as a biomarker for risk of future psychopathology in the adolescent brain, as well as being a predictive marker for prognosis and the degree of symptom severity following brain insults.

Our results only displayed maturational effect in one of six frontoparietal components, and thus revealed sparse age associations with the centrality of the frontoparietal network. Recent studies suggest that maturation of task positive networks may be better reflected by dynamic rather than static measures of functional connectivity, and that development as well as aging are accompanied by changes in dynamic transitions between brain states, operationalized through temporal variability in between-network connectivity (Ryali et al., 2016; Córdova-Palomera et al., 2017; Faghiri et al., 2017). Thus, graph and centrality measures based on a static apprehension of the brain connectome may fail to capture key aspects of maturational differences in the brain temporal dynamics.

The most prominent age-related reduction in centrality was seen in thalamus and cerebellum, converging with previous reports (Baker et al., 2015; Sato et al., 2015a; Petrican et al., 2017). Subcortical nodes have been reported to display enhanced connectivity with prefrontal nodes during adolescence (Ezekiel et al., 2013; Sato et al., 2015a). In conjunction, these findings suggest that subcortical networks reduce its influence throughout adolescence, through a drop in internal connectivity, shifting toward higher influence from frontal cortices and increased top-down control (Baker et al., 2015). Indeed, aberrant thalamo-cortical connectivity in adults have been linked to mental disorders (Wang et al., 2015; Skåtun et al., 2018), where the suggested mechanisms include altered inhibition of sensory signaling. In line with the notion that cognitive and emotional development during childhood and adolescence is accompanied and supported by substantial tuning and specialization of brain network activation and connectivity, it was recently demonstrated that the brain connectome develops into a more stable and individualized pattern, and that a delay in this development is associated with increased burden of mental health issues (Kaufmann et al., 2017a).The balance between maintaining a stable and idiosyncratic connectivity pattern across contexts on one hand and brain network flexibility in response to dynamic contextual changes on the other may reflect a key characteristic of a healthy mind. Delineating the maturational trajectories of dynamic shifts in hub properties between mental states may provide important clues for healthy and pathologic brain development, as well as shed light on adult neuronal pathology. Indeed, degree of damage to hub locations have been shown to be a key predictor of cognitive impairment (Warren et al., 2014).

### Limitations

The current study employed a cross-sectional design. This limits the conclusions that can be made, as there is no measure of within-person changes, and cohort effects cannot be ruled out. To further differentiate healthy and pathologic development, longitudinal studies will be of importance to better identify markers for delayed and aberrant maturation. Furthermore, the mounting evidence for misinterpreted motion-driven age effects in previous developmental studies on FC (Power et al., 2012), calls for caution when interpreting maturational findings. Although we implemented stringent procedures for data denoising (Kaufmann et al., 2017a), it is not possible to completely rule out residual motion effects as a confounder regarding age-related findings.

### Conclusion

Our study adds to the mounting evidence supporting the importance of hub development for healthy maturation during adolescence. The activation and centrality of cingulo-opercular components were associated with task engagement, task performance, as well as increasing age. This supports the importance of the hub properties of cerebellum and cingulo-opercular network as key to healthy development of executive capacities, as well as its vulnerability to injuries and deviant development.

## Synthesis

Reviewing Editor: Cheryl Sisk, Michigan State University Decisions are customarily a result of the Reviewing Editor and the peer reviewers coming together and discussing their recommendations until a consensus is reached. When revisions are invited, a fact-based synthesis statement explaining their decision and outlining what is needed to prepare a revision will be listed below. The following reviewer(s) agreed to reveal their identity: Kathryn Mills, Marie-Helene Grosbras.

Reviewers agree that your paper is well-written, methodologically sound, and provides new information on how brain regions change in centrality across adolescence, and how these changes relate to working memory performance. There are a few suggestions for clarification and revision. Please address these in a revised manuscript, which will be reviewed by me.

1. It is unclear why the authors had to resample the EC-maps back to 2mm to continue with the rest of the analysis.

2. The EC maps of three components were related to working memory task performance, yet it appears that one cingulo-opecular node is overshadowing the narrative of this manuscript. Could the authors unpack the cerebellum and bilateral thalamus findings a bit further?

3. Please provide more detailed figure descriptions, as some figures are difficult to understand without referring to the main text (e.g. figure 1 color bar needs labels; figure 3 needs clearer labeling).

4. Please consider uploading all statistical maps to NeuroVault for easier interpretability.

5. Will processing and/or analysis scripts be made available to facilitate reproducibility and replication efforts?

## Author Response

Response to the Reviewers:

Dear Dr. Cheryl L Sisk

Thank you for the positive and constructive feedback, and for the opportunity to submit a revised version of our paper “Key brain network nodes show differential cognitive relevance and developmental trajectories during childhood and adolescence” for consideration in eNeuro.

Below we address the comments: -It is unclear why the authors had to resample the EC-maps back to 2mm to continue with the rest of the analysis.

Response: The filtered and denoised fMRI datasets were resampled to 3 mm space due to the high computational demands of the voxelwise EC estimation. The main reason for the subsequent resampling of the EC maps from 3 mm to 2 mm isotropic space after EC estimation was convenience. The initial fMRI preprocessing and registration to MNI space included a sampling of the raw data to 2 mm MNI space. The common mask used during EC estimation is based on the individual normalized masks in 2 mm space, and the MNI 2 mm brain template was used as background for visualization and anatomical interpretation of the group ICA maps. A possible concern regarding resampling of the ECM maps to a higher resolution before further analysis is that the interpolation may influence the degrees of freedom and smoothness of the maps. Importantly, both ICA estimation on the EC maps, as well as the TFCE performed for the full brain voxel-vise analysis adapts to the smoothness of the images and it is highly unlikely likely that the resampling have influenced or biased the results.

The EC maps of three components were related to working memory task performance, yet it appears that one cingulo-opecular node is overshadowing the narrative of this manuscript. Could the authors unpack the cerebellum and bilateral thalamus findings a bit further?

Response: We thank the reviewer for these thoughtful comments, and the opportunity to expand the discussion regarding our results. To clarify our findings, we have now revised a section of the discussion, which now reads:

p. 14, line 326-328: “The most prominent finding in the current study was increased centrality of frontoparietal, cerebellar and cingulo-opercular components, and decreased centrality in sensorimotor and thalamic components”

p14, line 340-342: “Indeed, within-network visual and sensorimotor connectivity have been reported to decrease with task demand, with simultaneous increases in connectivity with frontoparietal and cerebellar nodes.”

p. 14 -- 15, line 344 -- 363: “Thalamus plays a key role in the gating of sensory input towards higher cortical network structures (Halassa and Kastner, 2017; Hwang et al., 2017). Interestingly, reduced connectivity between specific sub-clusters of the thalamus and visual and motor cortices in response to task demands has been reported, also in absence of altered FC with executive or DMN networks (Fan et al., 2015). In light of these observations, our findings indicate that task engagement reduces thalamic FC with cortical areas, facilitating the suppression and filtering of sensory signal during task engagement.

2

Our results align with previous findings highlighting the importance of increased influence of executive and cerebellar nodes during task engagement, as higher cingulo-opercular, cerebellar and frontoparietal centrality was associated with better task performance, and higher centrality in visual and sensorimotor nodes was associated with lower performance. Thalamus in particular was further associated with decrease in centrality during task engagement reflecting a drop in global FC, and the degree of differentiation between task and rest EC was positively associated with performance. Indeed, our results support both the interference-hypothesis (D‘Esposito et al., 1999), suggesting that performance during the n-back task is supported by increased influence from frontoparietal and cingulo-opercular nodes, providing top-down attentional control, as well as the sensory gating hypothesis (Cromwell et al., 2008), reflected through the decreased thalamic EC, which was associated with better performance.”

p. 18, line 444-447: “Indeed, aberrant thalamo-cortical connectivity in adults have been linked to mental disorders (Wang et al., 2015; Skatun et al., 2017), where the suggested mechanisms include altered inhibition of sensory signaling.”

Please provide more detailed figure descriptions, as some figures are difficult to understand without referring to the main text (e.g. figure 1 color bar needs labels; figure 3 needs clearer labeling).

Response: We apologize for the poor labeling and legends in the initial version of the manuscript. Please see the revised version of the manuscript for improved figure layout and legends.

Please consider uploading all statistical maps to NeuroVault for easier interpretability.

Response: We thank the reviewer for this suggestion. We have now uploaded the ICA decomposition of the ECM maps estimated on resting state and n-back data, as well as t-maps from randomise for task effects on ECM maps to NeuroVault, under the collection-name ICA-ECM in the PNC sample: https://neurovault.org/collections/ACBWDVSP/

Will processing and/or analysis scripts be made available to facilitate reproducibility and replication efforts?

fMRI analyses and ECM estimation was done using freely available tools (FSL and Lipsia). For subsequent statistical analysis we wrote scripts in Matlab R2014a (The MathWorks Inc., Natick, USA). All code is available from the authors upon request, and we are looking into ways to share the scripts online.
